# Adherence to routine use of pharmacological prophylaxis of heterotopic ossification after total hip arthroplasty: results from an Italian multicenter, prospective, observational survey

**DOI:** 10.1007/s10195-012-0180-4

**Published:** 2012-02-23

**Authors:** Michele Barbato, Ezio D’Angelo, Giuseppina Di Loreto, Angelo Menna, Alexander Di Francesco, Vincenzo Salini, Umberto Zoppi, Lino Cavasinni, Pancrazio La Floresta, Carlo Luca Romanò

**Affiliations:** 1 Deptartment of Orthopaedics and Traumatology, Ospedale G. Bernabeo, Ortona, Italy; 2 Università degli Studi di Chieti “G. d’Annunzio”, Bari, Italy; 3 Deptartment of Orthopaedics and Traumatology, Ospedale San Salvatore, L’Aquila, Italy; 4 Deptartment of Orthopaedics and Traumatology, Policlinico SS. Annunziata, Chieti, Italy; 5 Deptartment of Orthopaedics and Traumatology, Ospedale G. Mazzini, Teramo, Italy; 6 Deptartment of Orthopaedics and Traumatology, Ospedale Civile SS Immacolata, Sulmona, Italy; 7 Deptartment of Orthopaedics and Traumatology, Ospedale Cardarelli, Campobasso, Italy; 8 Dipartimento di Chirurgia Ricostruttiva e delle Infezioni Osteo-articolari, Istituto Ortopedico I.R.C.C.S. Galeazzi, Via Riccardo Galeazzi, 4, 20161 Milano, Italy

**Keywords:** Heterotopic ossification, Hip, Total hip arthroplasty, Prophylaxis, Prevention, Celecoxib, Italy

## Abstract

**Background:**

In spite of the proven efficacy of pharmacological prophylaxis of heterotopic ossification following total hip arthroplasty, its routine use is still debated, and no data are available regarding the adherence to its administration in clinical practice.

**Materials and methods:**

In this prospective, observational, multicenter study, 480 consecutive patients operated on for primary total hip arthroplasty during the year 2009 were followed radiographically for 12 months after surgery in order to assess the incidence of periprosthetic heterotopic ossification. Surgeons were free to choose whether to administer pharmacological prophylaxis, and were asked to keep a record of the duration of the prophylaxis (if used) or the reasons for not using it. To facilitate the statistical analysis, all of the participating centers agreed to use only one drug (celecoxib) that had already proven to be effective.

**Results:**

368 patients were administered celecoxib and 112 patients did not receive any prophylaxis. Reported reasons for not administering celecoxib prophylaxis were the surgeon’s opinion that prophylaxis was not needed on a routine basis (84/112 patients, 75%), previous history of gastrointestinal bleeding (17.8%), and concomitant cardiorenal pathologies (7.1%). The overall incidence of heterotopic ossification in the celecoxib-treated patients was 23% (no cases of Brooker grade 3 or 4 ossifications), compared to 55% in the untreated patients (Brooker grade 3 and 4: 8.9%). Multivariate analysis showed that celecoxib prophylaxis was the single most important variable when predicting the occurrence of heterotopic ossification.

**Conclusions:**

This study confirms the efficacy and tolerability of celecoxib for the prophylaxis of heterotopic ossification after total hip arthroplasty, and shows how the surgeon’s belief that routine prevention is not required still plays an important role in the determination of this complication, together with the fear of possible unwanted side effects.

## Introduction

Despite the evidence that heterotopic ossification (HO) can occur with an incidence ranging from 15 to 90% after conventional total hip arthroplasty (THA), and that about one-quarter of those patients will develop severe HO (Brooker [1] grades 3 and 4) which may be associated with impaired range of motion (ROM) at the hip joint and decreased functional outcome [2–5], a recent review article has suggested that routine HO prophylaxis is not warranted for routine THA [6], while it has been advocated for all surface replacements, given the higher incidence of complications compared to that for THA [7].

Several patient-related risk factors have been implicated in the development of HO after THA, such as age, male sex, hypertrophic osteoarthritis, ankylosing spondylitis, diffuse idiopathic skeletal hyperostosis, and history of HO [3, 8, 9]. However, since soft-tissue trauma is the main initiating factor in HO development [10, 11], and given the fact that HO may develop even in the absence of any known risk factor, the question of how to determine whether a patient should receive HO prophylaxis remains unresolved.

Low-dose irradiation after total hip arthroplasty has been reported to be effective in the prevention of HO [12, 13], but extensive use of irradiation is limited by logistic problems, costs, and concerns about irradiating a vast population of patients. On the contrary, nonsteroidal anti-inflammatory drugs like indomethacin provide easy-to-use and effective prophylaxis that can be administered in any hospital, even though side effects can limit its use [14–18]. As a valid alternative, celecoxib has been previously reported to be as effective as indomethacin but with fewer side effects [19], and to be better than ibuprofen [20].

In spite of this large body of evidence in favor of pharmacological prophylaxis, there is still a lack of consensus as to the need for extensive prevention of HO after THA.

The aim of this prospective, multicenter study was to observe adherence to the routine use of pharmacological prophylaxis of HO after primary THA in six orthopedic wards in two Italian central regions (Abruzzo and Molise), and to correlate that with the incidence of HO.

## Materials and methods

This study, performed under the aegis of GAMOT (Gruppo Abruzzo-Molise Ortopedici e Traumatologi), included 504 patients (126 males and 378 females) who were affected by hip osteoarthritis and were undergoing surgery to implant a cementless total hip arthroplasty on six orthopedic wards in Italy [Ospedale S. Salvatore, L’Aquila (AQ): 95 patients; Ospedale di Sulmona (SU): 86; Ospedale G. Bernabeo, Ortona (OR): 83; Ospedale di Teramo (TE): 83; Ospedale di Campobasso (CB): 82; Ospedale SS. Annunziata, Chieti (CH): 75] during the year 2009. Twelve (2.4%) of these patients were lost to follow-up and so were not included in this review.

All the patients gave their informed written consent to collect their data prior to being included into this prospective, observational, multicenter study. The study was authorized by the local ethical committee and performed in accordance with the ethical standards of the 1964 Declaration of Helsinki as revised in 2000.

Exclusion criteria were previous surgery on the same hip, ankylosing spondilitis, diffuse idiopathic skeletal hyperostosis, neurologic diseases—all conditions that are known or suspected to increase the risk of development of heterotopic ossification [21, 22]. No patient received radiotherapy after the THA implant for HO prevention.

Preoperative diagnosis, surgical approach (posterolateral or direct lateral), HO prophylaxis, and its side effects were evaluated by a local investigator on each orthopedic ward. Surgeons were left free to choose whether or not to administer pharmacological prophylaxis of HO on the basis of their experience and their clinical judgement, but for those patients who did not receive prophylaxis, the surgeons were asked to make a record of the reason for their choice. To unify the data in this observational study and allow further statistical analysis, all of the participating centers agreed to use only celecoxib for the pharmacological prophylaxis of HO.

Heterotopic ossification was evaluated by a radiologist blinded to the treatment the patient received. The grading of heterotopic ossification was performed using anteroposterior radiographs of the hip at 12 months after surgery, according to the classification of Brooker et al. [1]. This system classifies the absence of heterotopic bone formations as grade 0, the presence of islands of bone within the soft tissues of the treated hip as grade I, the occurrence of bone spurs and a gap between opposing bone surfaces of >1 cm as grade II, the presence of bone spurs and a gap of <1 cm as grade III, and a bridge of bone across the joint as grade IV. Heterotopic ossification of grade III or more is associated with increasing impairment of range of motion and function.

Patients for whom the treatment was stopped because of the occurrence of side effects were not included in the statistical evaluation of heterotopic bone formation.

To analyze sample characteristics, Fisher’s exact test was used for categorical variables or linear regression models for continuous variables. Statistical significance was defined as a *P* value of less than 0.05, and 95% CI.

## Results

At follow-up, 492 patients were available; 380 (77.2%) patients were administered pharmacological prophylaxis (celecoxib 200 mg twice per day, for 17 ± 3 days, a minimum of 14 and a maximum of 20 days after surgery, starting the day after the surgical procedure in all cases). Twelve (3.2%) patients treated with celecoxib reported minor gastrointestinal side effects that required treatment discontinuation after a mean of 9 days (range 7–15 days) from the start of treatment, and were not included in the review of HO any further. No patient received indomethacin.

**Fig. 1:**
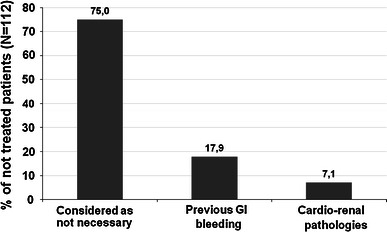
Surgeons self-reported reasons for not administering pharmacological prophylaxis in patients that underwent total hip replacement in this study (*N* = 112)

One hundred twelve (22.8%) patients did not receive any prophylaxis for HO; reported reasons for not administering prophylaxis were the surgeon’s opinion that prophylaxis was not needed on a routine basis (84/112 patients, 75%), a previous history of gastrointestinal bleeding (20/112; 17.9%), or concomitant cardiorenal pathologies (8/112; 7.1%) (Fig. 1).

**Fig. 2:**
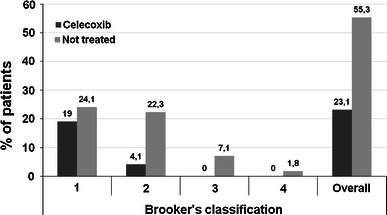
Incidence of heterotopic ossification in the celecoxib (*N* = 368) and in the untreated (*N* = 112) groups

The overall incidence of heterotopic ossification in the celecoxib-treated patients was 23.1% (85/368, no Brooker grade 3 or 4 ossifications), compared to 55.3% (62/112) in the untreated group. In the latter, 10/112 (8.9%) patients showed Brooker grade 3 and 4 ossification. Grades 1, 2, 3, and 4 HO were respectively seen in 27 (24.1%), 25 (22.3%), 8 (7.1%), 2 (1.8%) untreated patients; grades 1 and 2 HO were observed in 70 (19.0%) and 15 (4.1%) of the patients treated with celecoxib (Fig. 2). The overall difference in HO in the two groups (23 vs. 55%) was statistically significant (*P* < 0.0001).

Logistic regression showed that the occurrence of HO did not correlate with sex (*P* = 0.66), preoperative diagnosis (*P* = 0.14), hospital (*P* = 0.24), or surgical approach (*P* = 0.09), while celecoxib prophylaxis was the single most important variable in predicting the occurrence of heterotopic ossification (*P* < 0.0001; O.R.: 4.1; CI: 2.6–6.4).

## Discussion

There is a large body of evidence on the incidence of HO after THA; in the literature, it ranges from 15 to 90%, with the rate of severe HO (Brooker grades 3 and 4) at around 10%, in the absence of adequate prophylaxis [2]. Severe HO have been shown to be associated with impaired range of motion (ROM) at the hip joint and decreased functional outcome [3–5]. Even though several patient-related risk factors for developing ectopic ossification are well known [18, 22], as stated by Kolbl and Knelles et al. [13]: “on an absolute scale considerably more patients without risk factors develop heterotopic ossification because the number of patients with risk factors is low compared to all patients receiving total hip replacement. In this respect, prophylactic treatment after total hip replacement seems advocated for all patients.” Contrary to this statement, other authors have recently suggested that “there is currently little evidence to support the routine use of prophylaxis for heterotopic ossification in arthroplasty patients” [6], while pharmacological prophylaxis has been advocated for all surface replacements, given the higher incidence of the complication compared to THA [7]. More recently, HO prophylaxis has also been advocated after hip arthroscopy for femoroacetabular impingement syndrome [23].

This is, to our knowledge, the first prospective, observational, multicenter study ever performed to investigate the actual application of routine pharmacological prophylaxis after THA in patients without known risk factors for HO in different orthopedic wards of general hospitals in Italy and in Europe.

The incidence of HO observed in the present study in patients who were not treated with prophylactic measures and did not have any known risk factor is perfectly in keeping with those previously reported in the literature. Our study confirms the efficacy of administering celecoxib postoperatively to prevent this complication and the relatively low rate of side effects and dropouts connected with this prophylaxis. Our data also illustrate the reported reasons for not treating patients on a routine basis. In this regard, the belief that routine HO prophylaxis is not justified for patients without known risk factors for HO is the main reported cause for not administering pharmacological prophylaxis in our study. This may be due to the conflicting statements in the literature that are in favor of [13–20] or against the routine use of prophylaxis [6]. Other reported reasons for not performing pharmacological prophylaxis include risk factors for potential side effects connected with the use of anti-inflammatory drugs [24]. The importance of minimizing possible side effects in the clinical setting may also explain the universal preference of all the surgeons included in this survey for celecoxib rather than other possible nonsteroidal anti-inflammatory drugs. In fact, celecoxib has previously been reported to be equally effective but associated with fewer side effects than indomethacin [19, 25], while it has proven more effective than ibuprofen [20]. Considering the reasons for not administering pharmacological prophylaxis critically, in the light of the most recent reports on the safety of celecoxib used in association with proton pump inhibitors, even in patients with severe gastrointestinal risk factors [26, 27], it is questionable as to whether previous gastrointestinal bleeding should be considered a contraindication to pharmacological prophylaxis of HO with celecoxib. On the other hand, there is no clear evidence that short-term administration (less than 20 days) of celecoxib or any other nonsteroidal anti-inflammatory drugs can have serious side effects on the cardiorenal apparatus [28].

Limits of the present study include:

- The absence of a comparator group of patients treated with other nonsteroidal anti-inflammatory drugs
- The absence of a placebo control group
- The patient’s allocation to the two groups based on the surgeon’s choice
- The lack of complete information on comorbidities and their relative incidences
- The lack of information on possible subclinical side effects of the prophylaxis (e.g., the incidence of lower gastrointestinal tract bleeding)
- The lack of demonstration that prophylactic treatment with lower doses or a shorter duration would have been equally effective

In spite of these limitations, the present study confirms the efficacy and safety of pharmacological prophylaxis of HO with a selective cycloxygenase-2 inhibitor, and favors the routine administration of this prophylaxis after THA—even in patients without known risk factors for this complication, given the high incidence of complications in untreated patients.
